# Age-Based Tracks: A Method to Tailor Autism Diagnostic Evaluation in Large-Scale Autism Specialty Centers

**DOI:** 10.3390/jcm11216332

**Published:** 2022-10-27

**Authors:** Emily Fox, Jennifer Gerdts, Kaitlyn Ahlers, Beth Kotchick

**Affiliations:** 1 Seattle Children’s Autism Center, Seattle Children’s Hospital, Seattle, WA 98105, USA; 2 Department of Psychology, Loyola University Maryland, Baltimore, MD 21210, USA; 3 Department of Psychiatry and Behavioral Sciences, University of Washington, Seattle, WA 98195, USA; 4 Department of Psychiatry, Geisel School of Medicine, Dartmouth College, Hanover, NH 03755, USA

**Keywords:** autism, evaluation, age, tracks, COVID-19

## Abstract

This paper describes a proposed model of diagnostic evaluation for autism spectrum disorder (ASD) at a large-scale ASD specialty center. Our center has implemented age-based diagnostic tracks within an interdisciplinary team evaluation approach to assessing ASD. Data were collected as part of a program evaluation and included responses from provider surveys as well as patient medical record reviews. The results from 803 patients were included. The diagnostic outcomes, time for evaluation, and appropriateness of referral were analyzed in patients referred to the Younger (*n* = 155) and Older (*n* = 648) diagnostic tracks. In 92.8% of cases referred to the clinic’s standard team evaluation model, the provider teams were able to make a diagnostic decision within the allotted evaluation time. The results from an additional diagnostic pathway, termed the Autism Psych Team (APT), within the older track were also presented. The intake providers had the option to triage older patients to this pathway when they anticipated that the patient might be diagnostically complex. Most patients (45.1%) triaged to the APT were referred due to psychiatric complexity. In 96% of APT cases, the APT providers felt the patient was an appropriate referral. Overall, these results suggest a method to efficiently triage patients to diagnostic models equipped to serve them within a high-volume ASD center.

## 1. Introduction

Autism spectrum disorder (ASD) is a complex neurodevelopmental condition characterized by social communication differences and restricted and repetitive patterns of behavior [1]. ASD affects an estimated 1.5 million children in the United States (U.S.) and is found across all racial, ethnic, and socioeconomic groups [2]. ASD is a heterogeneous disorder, as it is diverse in its symptom presentation and severity and is also often accompanied by differences in cognitive, language, behavioral, and emotional functioning. The variability and nuances inherent to autism can make it challenging for many clinicians to diagnose. As such, children who present with ASD symptoms are often referred to specialists or specialty centers for further assessment [3,4,5]. Given that there is no medical test that can reliably and accurately identify ASD across individuals, diagnosis relies on a combination of clinical judgment, direct assessment of behavioral symptoms, and patient and caregiver reporting [6].

The path to an ASD diagnosis can be tumultuous for families. While parents often express early developmental concerns about their children who go on to be diagnosed with ASD [2], and skilled clinicians can reliably diagnose autism in early toddlerhood [7,8], the average child with ASD is not diagnosed until after the age of four [2]. One study [9] surveyed almost 500 parents of children with ASD and found that the children were typically seen by four to five clinicians for evaluations before receiving an ASD diagnosis. The older the child, the more providers the child saw before finally receiving a diagnosis [9].

There are several professional and structural factors that contribute to what has been referred to as the “diagnostic odyssey” in ASD [10], or the frequently lengthy period of time between when concerns are raised about a child’s development and when a diagnosis is ultimately provided [11]. These include lengthy and labor-intensive evaluation models [11], limited provider training and confidence in diagnosing ASD [11,12], and a limited number of available ASD specialists [12]. These factors, in addition to increased awareness of and attention to ASD, have contributed to long waitlists at ASD specialty centers, a clinical issue some experts have deemed a “crisis” in the ASD field [5].

To alleviate this backlog and increase much-needed access to specialty diagnostic services for families, there has been a push for creative service delivery models that increase the efficiency of evaluations without sacrificing quality of care [11]. A variety of ASD diagnostic evaluation models has been suggested and used in practice. These include single-discipline models, in which one provider, often a psychologist or physician, independently assesses a patient over the course of several appointments. Multidisciplinary evaluations, in which a team of providers from different disciplines assesses the same patient over multiple appointments while each provider stays within the purview of their respective discipline [13], are also common. Finally, an interdisciplinary team evaluation model, in which two or more clinicians of different disciplines conduct the diagnostic evaluation collaboratively, is a third approach to assessing ASD [14] and is the evaluation modality of interest in the current study. One large ASD specialty center on the west coast, the Seattle Children’s Autism Center (SCAC), uses an interdisciplinary approach to assess children referred for concerns about ASD, with the expressed goal of considering or ruling out an ASD diagnosis [14]. A program evaluation examining this model found that interdisciplinary teams were able to make a diagnostic decision (i.e., yes or no for ASD) in 90% of cases evaluated in a single day [14]. In comparison with the multidisciplinary teams at SCAC, the patients seen through interdisciplinary teams were more likely to return to the clinic for follow-up appointments, the interdisciplinary team providers reported greater professional satisfaction, and the interdisciplinary teams billed significantly fewer hours than the more common psychology-led multidisciplinary teams [14].

The team evaluation model has been revised and refined in subsequent years. The current paper presents the results from a follow-up program evaluation of the implemented changes at SCAC, including the creation of age-based diagnostic tracks and the addition of an “Autism Psych Team (APT)” evaluation model available for children with complex clinical presentations or histories. We present data on the referral trajectory from diagnostic intake to evaluation and further delineate this by the age-based track and evaluation format (i.e., in-person vs. telehealth). Our statistical analyses explore how the implemented changes to our model impact evaluation and clinical outcomes. We hypothesize, for example, that the availability of the APT model would help to decrease the percentage of patients referred to standard teams who need additional time for evaluation. We conduct analyses to determine whether there are differences in patient demographics (i.e., age, sex, race or ethnicity, use of a language interpreter, and insurance type) by team referral type (standard or psych). We also examine the rates of ASD diagnosis by the age-based track and by team evaluation type (standard or psych) and summarize the diagnoses commonly provided in the cases where ASD is not diagnosed. We expected that most patients referred to our center would ultimately be diagnosed with ASD, as this would be consistent with the findings from the initial program evaluation [14]. Finally, we were interested in identifying markers of appropriateness of the diagnostic track for evaluating a given patient. For example, we present data on provider opinion regarding the appropriateness of referral and time used to complete evaluations.

## 2. Materials and Methods

### 2.1. Interdisciplinary Team Evaluation Model Overview

Our autism center operates within a large pediatric hospital and is the largest autism specialty center by volume in the region. In recent years, we received an average of 1962 unique diagnostic referrals annually. The center employs 51 providers of different disciplines, 25 of whom work in our diagnostic clinic. Patients are referred for ASD evaluation at SCAC by both external and internal providers. Most referrals are external and originate from primary care providers throughout the state. Based on the findings from Gerdts et al. (2018), SCAC transitioned to an all-interdisciplinary-team model of diagnostic evaluation. In subsequent years, this process was refined in response to feedback from center clinicians, as well as additional program evaluation data [15]. Two primary changes were implemented. First, two age-based diagnostic tracks were created based on qualitative feedback from clinicians that the time involved in conducting evaluations often differs depending on the patient’s age (i.e., younger children often have less history to review, fewer diagnostic differentials, and sometimes have clearer symptom presentation). Thus, patients were triaged based on age to either (1) the “5 years and younger” (Younger) track or (2) the “6 years and older” (Older) track. Second, within the older track, we created two team evaluation pathways. Given pilot data from a small sample of patients evaluated in the Older track, which found that ~18% of patients required further information following their team evaluation in order for a diagnostic determination to be made [15], we added an “Autism Psych Team” (APT) track. This track was designed for children at least 6 years of age with complex clinical presentations or histories as determined by a diagnostic intake.

### 2.2. Components of Team Evaluation

#### 2.2.1. Diagnostic Intake

**Figure 1:**
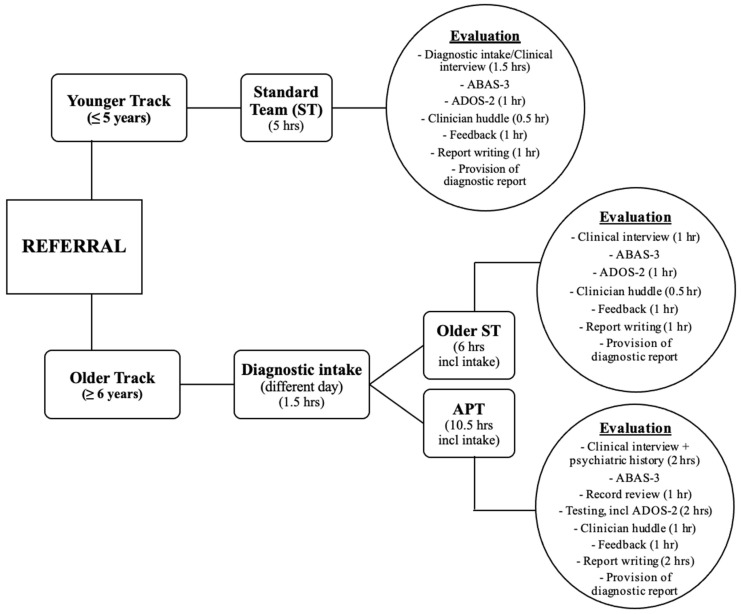
Team evaluation workflow and templated time, describing team evaluation components and templated time for each diagnostic model.

All children complete a diagnostic intake interview as part of their evaluation. In the Younger track, intake is conducted on the same day as the diagnostic evaluation appointment instead of as a standalone visit. For children in the Older track, the intake is completed in the weeks prior to the evaluation. Intakes are conducted by nurse practitioners (ARNPs), clinical psychologists, or physicians. The intake clinician gathers initial background information from the parent or caregiver about their concerns, as well as information regarding the child’s birth history and early development and the medical, psychiatric, and educational history. Intake providers also have the opportunity to informally observe the child over the course of the interview. For the Older children, based on the information gathered and the provider’s clinical impressions, the intake provider then decides which evaluation model—standard team (ST) or APT—is the most appropriate for the patient and makes the requisite referral. Below is an overview of the team evaluation models. See Figure 1 for a visual of this workflow, as well as the templated time for each track or model.

#### 2.2.2. Younger and Older Tracks: Standard Team (ST)

Both the Younger and Older children referred for a ST evaluation are seen by two clinical providers from different disciplines. The STs are made up of a combination of family practice and pediatric ARNPs, speech-language pathologists (SLPs), clinical psychologists, and physicians. Most (76.9%) pairings are psychologist-ARNP teams, followed by ARNP-SLP teams (14%) and psychologist-physician teams (8.9%). Evaluations consist of a diagnostic interview focused on ASD symptoms, ASD-specific testing using a structured, play-based assessment that includes modified activities from the Autism Diagnostic Observation Schedule, Second Edition (ADOS-2; [16]), collection of the Adaptive Behavior Assessment System, Third Edition (ABAS-3; [17]), a caregiver report measure of adaptive functioning, review of collateral reports and outside records (e.g., from schools or other providers), and an ASD-focused feedback session. One member of the provider team typically conducts the feedback, and the team collaboratively writes the diagnostic report.

#### 2.2.3. Older Track: Autism Psych Team (APT)

After intake of patients 6 years and older, providers may refer them to an APT when they anticipate a patient will be diagnostically complex based on clinical information obtained during intake. Unique to this model, APTs are conducted by providers who have specialized psychiatric training (i.e., clinical psychologists, psychiatrists, and psychiatric ARNPs). APTs include an ASD-focused clinical interview and review of psychiatric history. The ABAS-3 is collected, collateral records are obtained and reviewed, and the ADOS-2 is administered. There is additional time allotted in the evaluation template for neuropsychological testing or in-depth record review. As in the ST, one member of the provider team typically conducts the feedback, and the team collaboratively writes the diagnostic report.

#### 2.2.4. Telehealth

Consistent with other clinical programs across the U.S., the COVID-19 pandemic significantly disrupted ASD diagnostic services, given the reliance on face-to-face evaluations and using diagnostic tools traditionally administered in person [18]. During a portion of the time period captured in the current study, diagnostic services at SCAC had to be halted and then revised once resumed in response to the global COVID-19 pandemic. When evaluation appointments restarted, SCAC began conducting most diagnostic appointments apart from psychological testing via telehealth. Telehealth adaptations were made in consultation with other national autism centers (International Collaborative for Diagnostic Evaluation of Autism (IDEA) [19]). Testing was typically conducted in person with appropriate precautions (e.g., symptom screening, COVID-vaccinated staff, the use of personal protective equipment, and visitor and patient masking), although there was an option to conduct evaluations entirely via telehealth in certain cases. The results from the current study need to be understood within the context of these atypical assessment conditions.

### 2.3. Program Evaluation

SCAC initiated an program evaluation to monitor and evaluate the implementation of the age-based diagnostic tracks, as well as the newly created APT model. For the Younger children, patient outcomes were collected following the completion of their team evaluation. For the Older children, patient outcomes were collected at two time points: post-intake (Older Time Point 1) and post-evaluation (Older Time Point 2). The program evaluation procedures are described in detail below. This study was approved by the Institutional Review Board (IRB) with a waiver of informed consent and Health Insurance Portability and Accountability Act (HIPAA) authorization.

#### 2.3.1. Data Collection

The center’s clinic schedule was monitored daily by the analysis team to identify (1) Older patients who completed a diagnostic intake appointment (Older Time Point 1), (2) Younger patients who completed a team evaluation, and (3) Older patients who completed a team evaluation (Older Time Point 2). Appointment completion (i.e., either completion of a team evaluation for Younger children or an intake appointment for Older children) was the only inclusion criterion for the program evaluation, and no patients were excluded. For the Older children, intake providers indicated via a short survey (1) the type of evaluation referral that was to be submitted (ST, APT, or no evaluation) and (2) the initial diagnostic impression of the patient (ASD or no ASD). If the patient was being referred for an APT, the provider was asked to select one or more reasons for this from a list of the following options developed based on feedback from center clinicians: (1) significant psychosocial history, (2) multiple psychiatric concerns, (3) complicating intellectual functioning, (4) prenatal alcohol or drug exposure, or (5) other.

For all evaluations, the team providers completed a short survey about the patient’s diagnostic outcome, time required to complete evaluation, and access to records. In the cases where the providers specified that they needed additional time to reach a diagnostic conclusion, they were asked to provide a qualitative description of the reason for this. Specific to the APT, the providers were asked if, in their opinion, the patient was an appropriate referral for an APT or whether the patient could have been served by an ST. All provider responses were recorded in REDCap, a password-protected, HIPAA-compliant electronic database. If a provider did not respond to the survey, then the patient’s medical record was reviewed to extract the requested information.

#### 2.3.2. Patients

**Figure 2:**
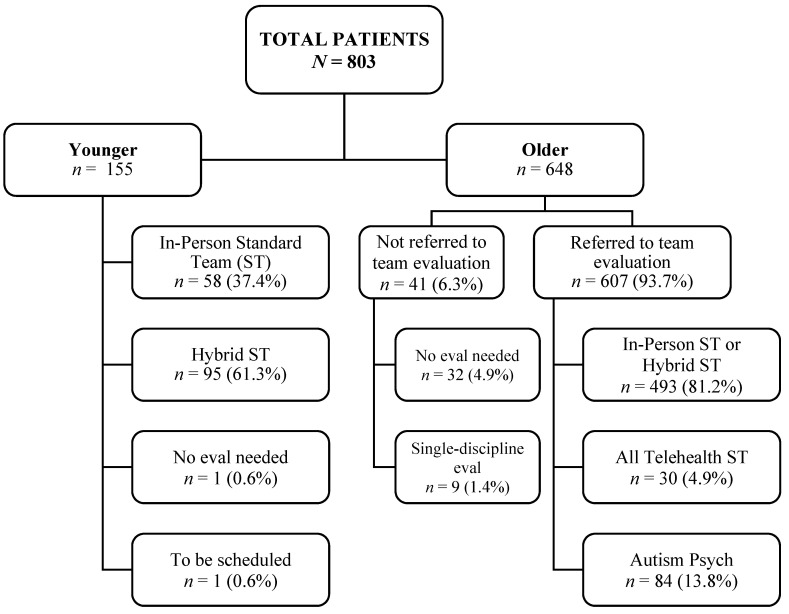
Evaluation referral tree, which summarizes how patients were triaged following diagnostic referral (Younger) and (Older) intake.

The current paper summarizes the data from 803 patients followed during the program evaluation time period between 2020 and 2022. This included 155 Younger patients who were triaged directly to team evaluation following their referral and 648 Older patients who first completed a diagnostic intake. See Figure 2 for a breakdown based on the referrals. Of note, an additional group of 151 children aged 4 years and younger was also followed during the program evaluation, while an entirely telediagnostic model of evaluation (i.e., use of the TELE-ASD-PEDS [20]) was piloted. The results from this group are described elsewhere because the evaluations were performed by a single discipline [21].

See Table 1 for the patient demographic data broken down by the age-based track and team model (ST vs. APT). In summary, the overall patient population ranged in age from 1.75 to 21 years (*M* = 9.5 years, *SD* = 4.2 years). Most patients (65.8%) were male. Of those patients with race and ethnicity data available, close to 44% self-identified as a person of color. About 7% of the patients used a language interpreter during their visits, and more than half (58.4%) had Medicaid or no insurance.

### 2.4. Statistical Analyses

The demographic variables of patient sex at birth, age in years at evaluation (Younger) or intake (Older), race, ethnicity, use of a language interpreter, and insurance type were used as covariates in all patient outcome analyses. Chi-square analyses and logistic regressions were used to examine the patients’ demographic variables and diagnostic outcome by team referral type (ST vs. APT) and age-based track.

## 3. Results

### 3.1. Evaluation Trajectory

#### 3.1.1. Younger Group

Following referral, 155 Younger children were sent for team evaluation. Of the 153 Younger children whose evaluations were complete, 37.9% were seen in-person for all of their diagnostic appointments (in-person ST), and 62.1% were seen for a combination of in-person and telehealth appointments (hybrid ST). No significant differences in demographics (i.e., age, sex, race or ethnicity, use of an interpreter, and insurance type) were observed based on the evaluation format (*p* ≥ 0.225).

#### 3.1.2. Older Group

Of the 648 Older children seen for intake, 95.1% (*n* = 616) were referred for diagnostic evaluation. In the remaining 4.9% of cases (*n* = 32), the intake provider was either able to make a diagnostic decision at the conclusion of the intake or did not refer the child for further evaluation due to patient preference or the discovery of an already established ASD diagnosis. In total, 607 Older children were referred for team evaluation (the remaining 9 out of 616 Older children were seen in a single-discipline model). Most (86.2%) of these patients were referred to an ST. The remaining 13.8% of patients were referred to an APT.

Of the 492 Older ST evaluations that were completed or scheduled, 20.9% were in-person evaluations (in-person ST), and 73% involved a combination of in-person and telehealth appointments (hybrid ST). A small portion of these patients (6.1%) completed or were referred for their entire diagnostic evaluation via telehealth (all telehealth ST). Patients seen entirely in person were more likely to use an interpreter for their visits (X^2^(2) = 18.2, *p* < 0.001). Otherwise, no significant differences in demographics were observed among the in-person, hybrid, and all telehealth STs (*p* ≥ 0.376). Given that these three team types are reserved for non-clinically complex patients and follow a similar evaluation format, these referral groups were merged for the purpose of subsequent analysis, and they are referred to collectively as the Older Standard Team (Older ST).

A logistic regression was performed to assess the effects of demographic variables on the team referral type for Older patients. The model was statistically significant (X^2^(5) = 58.82, *p* < 0.001). Patient age and sex were significantly associated with team referral type (Older ST vs. APT). Girls were more likely to be referred to an APT than to an ST (*p* = 0.003), and an increasing age was associated with an increased likelihood of referral to an APT (*p* < 0.001). Race or ethnicity, use of an interpreter, and insurance type did not significantly contribute to the model (*p* ≥ 0.232).

The reasons for referral to an APT were examined. As the providers could select more than one reason for referral, these categories are not mutually exclusive. 37% of patients had more than one APT referral reason. The most common reason for referral to an APT was concern for a complex psychiatric differential (45.1%). For example, this included patients presenting a history of multiple mental health diagnoses, treatment in psychiatric residential or inpatient facilities, suicidal ideation or self-harming behaviors, disruptive or aggressive behaviors, substance abuse, gender dysphoria, or perceptual disturbances. A complex psychosocial history was the next most common reason for referral (21.6%) and included patients who had experienced abuse or neglect, sexual assault, homelessness, institutional care, or legal issues. Concerns about cognitive functioning or known cognitive impairment were a frequent reason for referral (12.7%). Patients with complicated medical histories made up 11.8% of APT referrals. This included patients with genetic changes known to be associated with ASD, congenital abnormalities, chronic health conditions, or physical limitations (e.g., being wheelchair bound or a tracheostomy tube). Finally, patients who had been exposed to illicit drugs or alcohol in utero represented 8.8% of APT referrals. 

### 3.2. Diagnostic Outcome

At the time of analysis, 663 team evaluations had been completed across all tracks and team models (153 Younger, 463 Older ST, and 47 APT). Several Older patients were lost to follow-ups after intake or were still waiting to be scheduled. About 73.2% of all children evaluated as part of the program evaluation received an ASD or provisional ASD diagnosis (72.8% of Older children compared with 74.6% of Younger children). There was ~72% agreement in the providers’ diagnostic impressions at intake with the diagnostic outcomes upon evaluation. In most cases of discrepancy, the intake providers expected the patient would receive an ASD diagnosis, but ASD was not ultimately diagnosed at evaluation (59.1%). There were no significant differences in diagnostic outcome based on patient age, sex, race or ethnicity, use of an interpreter, or insurance type (*p* ≥ 0.088). The evaluation format (telehealth vs. in-person vs. hybrid) also did not impact the likelihood of ASD diagnosis (*p* = 0.717). The rate of ASD diagnosis was compared further in the Older track by team type (Older ST vs. APT). About 72% of the patients evaluated via an ST received an ASD diagnosis, compared with about 79% of the patients evaluated via an APT (*X*^2^(1) = 0.93, *p* = 0.336).

The diagnostic outcomes were examined in the 168 cases in which ASD was not diagnosed (diagnostic outcomes were not mutually exclusive). In the Younger group, alternative diagnoses included global or other specified developmental delays (41.7%), attention-deficit/hyperactivity disorder (ADHD, 5.6%), and speech and language delay or disorders (33.3%). For the Older children who completed an ST, the most common non-ASD diagnoses provided or maintained by history included ADHD (28.5%), anxiety (18.2%), other specified developmental delays or intellectual disabilities (6.7%), speech-language disorders (6.7%), and behavior disorders (6.1%). As for the APTs, the most common non-ASD diagnoses provided or maintained included anxiety (19.2%), ADHD (15.4%), trauma- and stressor-related disorders (11.5%), and speech and language disorders (11.5%). See Table 2 for other diagnoses provided or maintained.

In a small number of cases across the Younger and Older tracks (*n* = 33), a diagnostic decision was deferred. These patients were asked to return to the clinic at a later date (e.g., after assessment and treatment of other mental health concerns or after additional time had passed for the patient to age and develop) for re-evaluation. The patients for whom a diagnostic decision was deferred were significantly younger on average than the patients who received diagnostic decisions (ASD or no ASD) at the conclusion of their evaluations (*F*(2657) = 6.15, *p* = 0.002). No significant differences were observed between groups based on sex, race or ethnicity, use of an interpreter, or insurance type (*p* ≥ 0.128).

### 3.3. Assessment of Diagnostic Track Structure

Overall, 7.2% of the Younger and Older ST evaluations needed more time than was originally scheduled to reach a diagnostic conclusion. The combined patients across both ST tracks who needed additional time were significantly older on average (*M* = 10.2 years, *SD* = 4.2 years) than the patients whose evaluations were completed in the standard amount of time (*M* = 8.4 years, *SD* = 4.1 years) (*t*(498) = −2.63, *p* = 0.009). No significant differences were observed between these two groups based on other demographic factors (*p* ≥ 0.323).

This outcome was further examined by age-based diagnostic track. In the Younger group, 1.4% of evaluations required more time, and 9.4% of evaluations in the Older ST group required extended time. Most commonly, this was due to a need for additional records, such as the need for teacher reports (*n* = 12). Other reasons included the need for cognitive testing to clarify the patient’s intellectual functioning (*n* = 8), multiple psychiatric concerns (*n* = 8), a complex psychosocial history (*n* = 4), unclear ASD symptom presentation (*n* = 4), the lack of patient participation in testing (*n* = 3), a complex medical history *(n* = 1), and discrepancies between the parental report and clinician observations (*n* = 1).

In 96% of the APT cases for which provider response data were available, the APT team felt the patient was an appropriate referral to the team type. For example, one provider, in referencing the multi-layered complexity of many APT patients, said “This patient had a complex psychosocial and medical history, including prenatal exposures, neglect, adoption, bilingual family, and concussion history.” Qualitative feedback from the APT providers indicated that the APT cases frequently required a lengthy record review in order to systematically understand the patients’ presentation and symptoms over time. As such, APT providers reported that the additional time templated for APT evaluations was most often used for record review rather than for additional testing.

## 4. Discussion

The wait times for ASD evaluation at specialty centers have become prohibitive and increase families’ stress during the evaluation process. One factor contributing to the “waitlist crisis” [5] is the in-depth nature of many current ASD evaluation models. This has led some large centers to re-examine their diagnostic models and focus on the core components of ASD evaluation to more efficiently evaluate patients while still providing quality clinical care. While this approach is important for improving access to diagnostic services, it is unrealistic to expect that a disorder as variable and complex as ASD can be evaluated using a one-size-fits-all diagnostic model. At the same time, conducting in-depth, individualized evaluations for every child is not feasible in large-scale organizations with immense waitlists. Streamlined, balanced systems are needed to address inefficiencies in healthcare and to ultimately decrease wait times for families [11].

### 4.1. A Tailored Approach to ASD Diagnostic Evaluation

The current paper examined a proposed “middle ground” between individualizing comprehensive evaluations for each patient and using a single approach for all diagnostic referrals. We created diagnostic tracks within our interdisciplinary team evaluation model using objective information (age) that would be straightforward for patient support staff and clinic schedulers to follow based on a set rule. Following referral, patients aged 5 years and younger at our center are now triaged to the Younger track, and patients 6 years and older are triaged to the Older track. The model has been further refined over time. For example, the template for Younger patients was shortened based on provider feedback that they were not using the entire allotted time for evaluation in this age group. Now, Younger children complete a diagnostic intake as part of their team evaluation appointment rather than as a separate appointment, as is the case for Older patients. This change in the evaluation template has allowed for more patients in the Younger group to be scheduled and has also shortened the wait time for these patients by several months [15].

Another important change to the team evaluation format has been the addition of an Autism Psych Team, which was created for patient populations within the Older group that ASD-specialized clinicians have found to be challenging to assess (e.g., adolescent girls without intellectual disabilities or patients with a history of multiple psychiatric diagnoses). The APT model is similar to traditional psychology-led evaluations, with the addition of a psychiatric medical provider in the evaluation team. The APT model allows additional time for testing, questionnaires, and diagnostic interviewing.

### 4.2. Primary Results

With the current study, we aimed to examine the changes made to the interdisciplinary team evaluation model, and our results suggest these changes have been effective. Despite shortening the Younger team evaluation template, the providers were still able to make diagnostic decisions within the allotted time in almost 99% of the Younger cases while also maintaining an ASD diagnostic rate similar to that of the Older group and rates from previous years at our center [14]. Following intake, 86% of the Older children were sent to STs, and of these, only 9.4% needed additional time or information, suggesting that the focused evaluation model and time allotted in the ST template were sufficient to make a diagnostic determination in the vast majority of cases. Of the ~14% of patients sent to the APT from intake, 96% were identified as appropriate referrals by the APT providers. Together, these data suggest that the patients were effectively triaged.

Our model uses a flexible approach that varies based on the patient’s characteristics such that additional time is available for patients who need it. This model is a promising approach to evaluation that efficiently triages patients to interdisciplinary tracks that vary in time and provider discipline based on the patient’s needs and clinical characteristics. Importantly, this model was successfully implemented in a large-scale autism specialty center with many providers of different disciplines. The center support staff and schedulers have successfully been able to follow our age-based guidelines for scheduling and provider templates, which is essential given the nearly 2000 diagnostic referrals our center receives and processes each year. This model also allows us to maximize the array of professional disciplines at our center by targeting patients that best fit a particular discipline’s clinical expertise (e.g., the use of SLPs in team evaluations with younger children who are more likely to present co-occurring language disorders or the use of psychiatrists in APTs to see older children and adolescents more likely to present co-occurring psychiatric disorders and psychotropic medications).

### 4.3. Diagnostic Outcomes

Of the patients evaluated over the course of the program’s evaluation, 73.2% received an ASD diagnosis. In the cases in which ASD was not diagnosed, other concerns often were present, including ADHD, anxiety and mood disorders, behavioral disorders, developmental delays, and speech differences. Trauma- and stressor-related disorders were diagnosed not only in more psychiatrically complex patients evaluated in an APT but also in patients triaged to the Older ST track.

### 4.4. Autism Psych Team

Interestingly, the rates of ASD diagnosis were similar between the Older patients seen via an ST and those seen via an APT. One APT provider offered that, in her experience on these teams, the ASD diagnosis often is fairly clear, and it is intense psychiatric comorbidity that requires extensive record review to clarify and understand in the context of the developmental history and ASD symptomology. It is possible that acute mental health concerns may have overshadowed or taken precedent over ASD symptoms in these cases during care by other mental health professionals. The most common reason for APT referral was due to the presence of multiple psychiatric concerns. The vast majority of these children had previously spent time in inpatient psychiatric units and were referred for ASD evaluation following discharge. At least one study [22] examining pediatric hospitalizations has found that children with ASD are hospitalized more frequently for psychiatric reasons than children without ASD. The presence of multiple psychiatric concerns and psychiatric hospitalization also may contribute to delays in ASD diagnosis [23]. This may, in part, provide context for why the children referred to APTs were found to be older on average than the patients referred to STs.

In addition to age, sex at birth was also found to be associated with team referral type, and girls were more often referred to APTs. This likely reflects longstanding difficulties in the field with accurately identifying autistic girls and women [24,25,26,27,28]. Girls with ASD are diagnosed later on average than boys [29]. ASD symptoms are thought to be presented in a unique or more subtle way in girls [25,27], which often leads to them being missed or overlooked [25,26]. Failing to accurately identify and diagnose ASD in girls and women comes at a high cost: autistic women are at increased risk of mental health concerns [30] and attempting suicide [31]. Many of the girls seen via APTs had been hospitalized for psychiatric reasons, including issues related to suicidality, and had other co-occurring mental health concerns.

Importantly, the APT provider mentioned previously explained that her team has found that providing an autism diagnosis seems to give clarity for why these patients have “really struggled and nothing has really helped them...it’s because we missed this piece along the line.” It is possible that with specialized diagnostic pathways such as our APT, which has been shown to be well-equipped to serve patients with complex needs, these patients can be more readily identified, diagnosed, and then referred to appropriate treatment resources and services.

### 4.5. Limitations and Future Directions

There were several limitations to this study. First, this was an examination of the clinic processes in place at SCAC and was not part of a controlled research study. As such, the patients could not be randomly assigned to diagnostic models (Older ST vs. APT). The intake providers made referral decisions based on information gathered during the diagnostic interview and clinical judgment. In addition, the clinical data in this research study were limited to demographics and diagnostic outcomes due to how patient information was stored in the electronic medical records. We were not able to access waitlist data for Older patients for the current program evaluation. This has been a persistently difficult variable to systematically extract from patients’ medical records. However, we are continuing to explore methods of data extraction and metrics, and we hope to be able to capture this essential variable in the future. It will also be important for future research to examine how changes to diagnostic evaluation models affect clinic revenue, as well as patient and provider satisfaction.

Due to the COVID-19 pandemic affecting the ability to conduct in-person visits as well as staffing limitations, only a small number of APTs was completed at the time of analysis. As COVID-19 precautions have eased, and the option for in-person evaluation has become more regular, APT-referred patients have been scheduled at increasing rates. Future research aims to continue to better define this group of patients, including attempting to operationalize and empirically validate markers of diagnostic complexity, with the goal being to continue to improve the ability to triage patients from diagnostic intake to diagnostic models that are best equipped to serve them. 

The COVID-19 pandemic created a natural experiment and foray into telehealth diagnostic evaluations for autism. In our evaluation, there were no differences in diagnostic outcomes across variations in evaluation format (i.e., telehealth vs. in-person vs. hybrid). Virtual or hybrid models have the potential to increase access to diagnostic evaluation for those living in rural areas and for underserved communities [32]. Therefore, continued innovation to create valid yet flexible methods for ASD evaluation, including those that incorporate telehealth and remote assessment, is critical [18]. As with other diagnostic models, there will be some patients for whom remote or hybrid models work well and others for whom they will not, and future research should consider for whom various modalities are most appropriate.

## 5. Conclusions

This study describes an effective interdisciplinary team evaluation model for assessing autism spectrum disorder at a large-scale autism center. In this model, patients referred for diagnostic evaluation are triaged to age-based diagnostic tracks for evaluation. The model also includes a diagnostic pathway for patients with complex clinical histories or backgrounds. The results from this research suggest this model is a promising approach to evaluation that can be successfully implemented within a high-volume clinic while also prioritizing patients’ needs and clinical presentation.

## Figures and Tables

**Table 1:** Patient demographic data by age group and referral type, describing patient demographic data.

	Younger (*n* = 155)	Older (*n* = 648)		Total (*n* = 803)
		**ST** **(*n* = 523)**	**APT** **(*n* = 84)**	
Age in Years: Range	1.75–5	6–20	6–21	1.75–21
Mean	3.8	10.4	13.4	9.5
SD	1.0	3.4	3.1	4.2
Sex at Birth (%)				
Male	75.5	66.3	45.2	65.8
Female	24.5	33.7	54.8	34.2
Race (%)				
American Indian and Alaskan Native	1.4	2.4	3.7	2.3
Asian	14.5	4.8	6.1	7.3
Black or African-American	13.8	4	4.9	6
Native Hawaiian and Other Pacific Islander	3.4	1.6	1.2	1.8
White	41.4	65.7	64.6	60.9
Other	17.9	11.1	8.5	12.2
Multiracial	7.6	10.5	11	9.6
Ethnicity (%)				
Non-Hispanic	77.6	82.5	86.7	81.9
Hispanic	22.4	17.5	13.3	18.1
Insurance (%)				
Commercial	40.9	42.1	36.9	41.6
Medicaid or No Insurance	59.1	57.9	63.1	58.4
Use of Interpreter (%)				
Yes	12.1	5.7	6	7.3
No	87.9	94.3	94	92.7

**Table 2:** “No ASD” diagnoses provided or maintained by history, listing diagnoses provided in the cases where ASD was not diagnosed. Diagnoses were not mutually exclusive, as patients could receive more than one diagnosis.

	Younger	Older: ST	Older: APT
	%	%	%
ADHD	5.6	28.5	15.4
Anxiety	-	18.2	19.2
Depression	-	4.2	7.7
Disruptive mood dysregulation disorder or bipolar disorder	-	0.6	7.7
Trauma- and stressor-related disorders	-	1.8	11.5
Global developmental delay or intellectual disability	-	6.7	3.8
Learning disability	-	1.8	3.8
Speech and language disorders	33.3	6.7	11.5
Selective mutism	-	0.6	3.8
Behavior disorders	-	6.1	7.7
Adjustment disorder	-	1.2	-
Sensory processing disorder	-	2.4	-
Tic disorder	-	0.6	-
Neurobehavioral disorder associated with prenatal alcohol exposure	-	-	3.8
Other specified neurodevelopmental disorder associated with a genetic change	-	-	3.8
Other specified developmental disorder or delayed milestones	41.7	20.6	-

## Data Availability

The data are not publicly available due to patient privacy.
